# Screening and identification of five serum proteins as novel potential biomarkers for cured pulmonary tuberculosis

**DOI:** 10.1038/srep15615

**Published:** 2015-10-26

**Authors:** Chong Wang, Li-Liang Wei, Li-Ying Shi, Zhi-Fen Pan, Xiao-Mei Yu, Tian-Yu Li, Chang-Ming Liu, Ze-Peng Ping, Ting-Ting Jiang, Zhong-Liang Chen, Lian-Gen Mao, Zhong-Jie Li, Ji-Cheng Li

**Affiliations:** 1 Institute of Cell Biology, Zhejiang University, Hangzhou 310058, P.R. China; 2 Department of Respiratory Medicine, The Sixth Hospital of Shaoxing, Shaoxing 312000, P.R. China; 3 Department of Clinical Laboratory, Zhejiang Hospital, Hangzhou 310013, P.R. China; 4 Department of Tuberculosis, The First Hospital of Jiaxing, Jiaxing 314001, P.R. China

## Abstract

Rapid and efficient methods for the determination of cured tuberculosis (TB) are lacking. A total of 85 differentially expressed serum proteins were identified by iTRAQ labeling coupled with two-dimensional liquid chromatography-tandem mass spectrometry (2D LC-MS/MS) analysis (fold change >1.50 or <0.60, *P* < 0.05). We validated albumin (ALB), Rho GDP-dissociation inhibitor 2 (ARHGDIB), complement 3 (C3), ficolin-2 (FCN2), and apolipoprotein (a) (LPA) using the enzyme-linked immunosorbent assay (ELISA) method. Significantly increased ALB and LPA levels (*P* = 0.036 and *P* = 0.012, respectively) and significantly reduced ARHGDIB, C3, and FCN2 levels (*P* < 0.001, *P* = 0.035, and *P* = 0.018, respectively) were observed in cured TB patients compared with untreated TB patients. In addition, changes in ALB and FCN2 levels occurred after 2 months of treatment (*P* < 0.001 and *P* = 0.030, respectively). We established a cured TB model with 87.10% sensitivity, 79.49% specificity, and an area under the curve (AUC) of 0.876. The results indicated that ALB, ARHGDIB, C3, FCN2, and LPA levels might serve as potential biomarkers for cured TB. Our study provides experimental data for establishing objective indicators of cured TB and also proposes potential markers for evaluating the efficacy of anti-TB drugs.

Pulmonary tuberculosis (TB) is a chronic infectious disease caused by *Mycobacterium tuberculosis* (Mtb). According to the World Health Organization (WHO) report, there were 9 million patients newly diagnosed with TB and 1.5 million deaths from TB in 2013 worldwide. In 2013, China reported 980,000 new TB cases and 41,000 TB deaths, accounting for 10.89% of the incident cases^1^. Thus, TB remains a major infectious disease in China.

Directly observed treatment short-course (DOTS) is recommended by the WHO as the strategy to cure TB. The treatment of newly diagnosed TB includes a 2-month intensive phase followed by a 4-month continuation phase^2^. At present, clinicians rely on the success of anti-TB therapy combined with chest X-ray, computerized tomography (CT) scans, sputum smears, and sputum culture tests to determine whether the TB patient is cured. Approximately 14% of discharged patients are not fully cured due to the lack of a uniform standard method for the determination of cured TB, increasing the risk of TB spreading. These uncured patients are vulnerable to relapse and may even develop multi-drug resistant TB^1^,^3^. The lack of a uniform standard method for the determination of cured TB may also lead to over-treatments of some TB patients, causing the augmentation of drug-related mortality^4^. Moreover, chest X-rays and CT scans are subjective tests, and sputum smear cannot distinguish dead from viable bacteria (sensitivity = 19.6%, specificity = 87.0%)^5^, and sputum cultures require 4 to 8 weeks of incubation time (sensitivity = 91.8%, specificity = 57.8%)^6^. Therefore, there is an urgent demand to establish a rapid and efficient method to determine cured TB^7^,^8^. Nahid *et al.*^9^ used the SOMAscan proteomics method and found differential changes in serum extracellular matrix protein 1, the tyrosine-protein kinase Yes, insulin-like growth factor-binding protein 1, cathepsin Z, coagulation factor V and serum amyloid A proteins between TB patients with 2 months treatment and untreated TB patients. Moraes *et al.*^10^ found that serum copper levels and the C-reactive protein/serum albumin ratio might be important indicators of TB treatment. IFN-γ-inducible protein 10 and pentraxin 3 are also significantly decreased in cured TB patients compared with untreated patients (*P* < 0.0001)^11^, indicating that these proteins could serve as biomarkers for cured TB. The above studies aimed at identifying biomarkers for treated TB, but they failed to establish a cured TB model.

In this study, differential serum proteins were screened using iTRAQ labeling coupled with two-dimensional liquid chromatography-tandem mass spectrometry (2D LCMS/MS) to identify potential biomarkers for cured TB. The candidate protein biomarkers were validated as predictors of anti-TB treatment outcomes. Our study provides experimental data for establishing an objective indicator of cured TB. It also provides potential markers for evaluating the efficacy of anti-TB drugs.

## Results

### Identification and relative quantification of serum proteins

**Figure 1:**
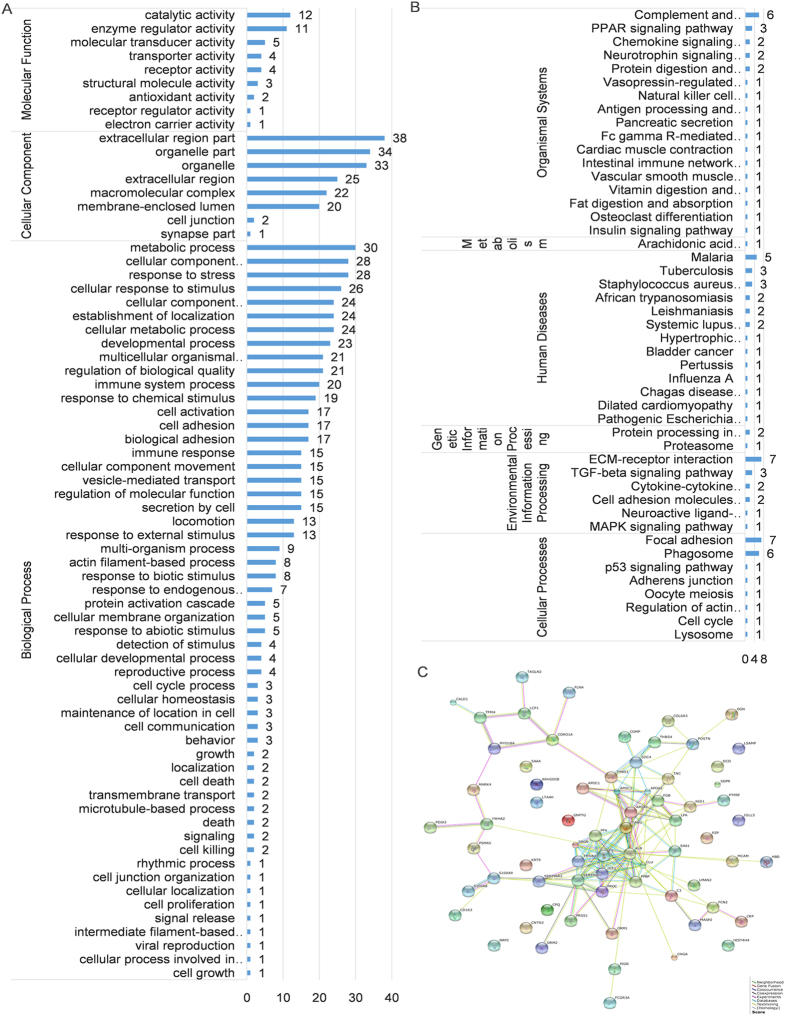
Data mining of the set of cured versus untreated tuberculosis serum biomarker candidates. (**A**) GO analysis; (**B**) KEGG pathway analysis; and (**C**) String analysis.

We identified 85 differentially expressed proteins by iTRAQ labeling coupled with 2D-LC MS/MS in cured patients and untreated smear-positive TB patients. Further screening revealed that 51 differentially expressed proteins were up-regulated (>1.50-fold, *P* < 0.05), whereas 34 proteins were down-regulated (<0.60-fold, *P* < 0.05) (Supplementary Table S1). Most of the differentially expressed proteins were involved in the metabolic process (30, 5.57%), cellular component organization or biogenesis (28, 5.19%), and response to chemical stimulus (28, 5.19%). In addition, these proteins were located in the extracellular region (38, 21.71%) and the organelles (34, 19.43%) and possessed catalytic activity (12, 27.91%) and enzyme regulator activity (11, 25.58%) (Fig. 1A). In addition, the proteins were also assembled in the following pathways: focal adhesion (7 proteins), extracellular matrix (ECM)-receptor interaction (7 proteins), complement and coagulation cascades (6 proteins), and phagosomes (6 proteins) (Fig. 1B). Protein-protein interactions were noted among all the proteins (Fig. 1C). We narrowed these 85 proteins to 35 proteins by adding a control group. Proteins down-regulated in untreated TB patients and up-regulated in the cured TB patients (Table 1), and proteins up-regulated in untreated TB patients and down-regulated in the cured TB patients (Table 2) were chosen.

### Enzyme-linked immunosorbent assay (ELISA) validation

**Figure 2:**
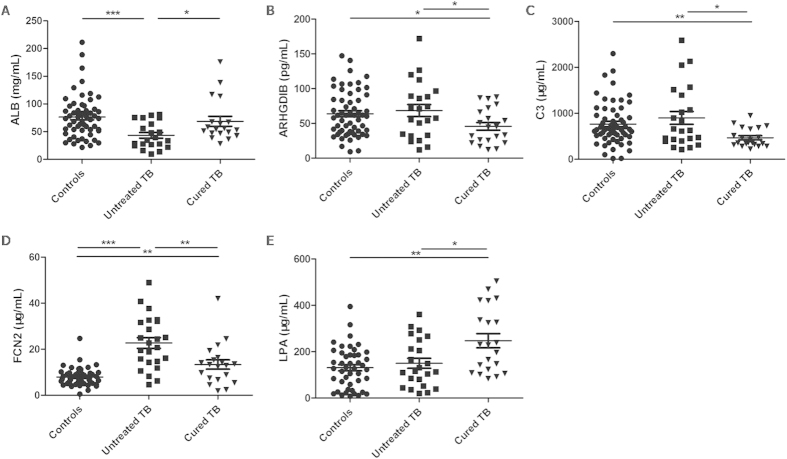
Serum proteins levels among the controls, untreated tuberculosis patients, and cured tuberculosis patients. TB: tuberculosis. A *P*-value less than 0.05 indicates statistical significance using the Mann-Whitney U-test. ^*^P < 0.05, ^**^P < 0.01, ^***^P < 0.001.

Based on the fold changes and the correlations with TB pathogenesis, we selected serum albumin (ALB, P02768, fold change = 8.11, *P* < 0.05), Rho GDP-dissociation inhibitor 2 (ARHGDIB, P52566, fold change = 0.28, *P* < 0.05), complement C3 (C3, P01024, fold change = 0.40, *P* < 0.05), ficolin-2 (FCN2, Q15485, fold change = 0.56, *P* < 0.05), and apolipoprotein (a) (LPA, P08519, fold change = 3.98, *P* < 0.05) for further verification. ELISA was performed among 25 untreated smear-positive TB patients, 23 cured TB patients (smear-positive when untreated), and 60 healthy controls. Significant differences in ALB, C3, FCN2, and LPA levels were noted among all three groups (*P* < 0.001, *P* = 0.011, *P* < 0.001, and *P* = 0.010, respectively). The results also revealed significantly increased serum ALB and LPA levels (*P* = 0.025 and *P* = 0.021, respectively), and significantly reduced ARHGDIB, C3, and FCN2 levels (*P* = 0.049, *P* = 0.034 and *P* = 0.003, respectively) in cured patients compared with untreated TB patients. In addition, significant differences were also noted in the ALB and FCN2 levels between untreated smear-positive TB patients and controls (*P* < 0.001 and *P* < 0.001, respectively) (Fig. 2). Significant differences in ALB, ARHGDIB, C3, FCN2, and LPA levels were also observed between cured and untreated TB patients (*P* = 0.036, *P* < 0.001, *P* = 0.035, *P* = 0.018, and *P* = 0.012, respectively) after including smear-negative TB patients. Moreover, significant differences in ALB, ARHGDIB, and FCN2 levels were noted between untreated TB patients and controls (*P* < 0.001, *P* = 0.003, and *P* < 0.001, respectively), whereas no significant differences were observed in ARHGDIB and C3 levels (*P* > 0.05).

**Figure 3:**
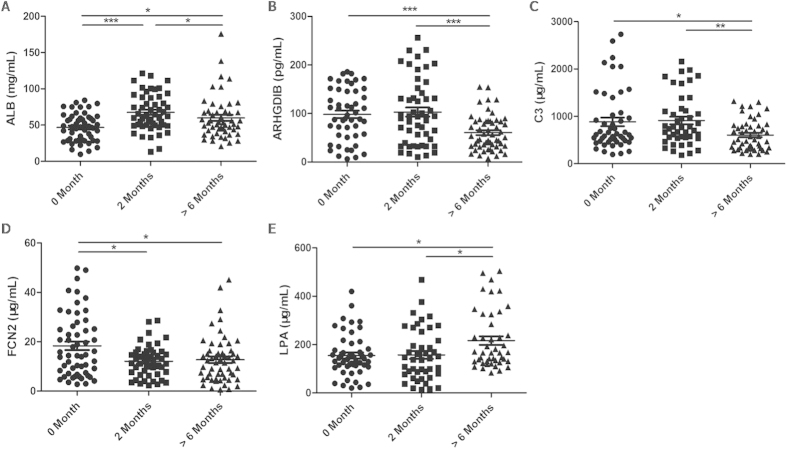
Serum proteins levels during tuberculosis therapy. A *P*-value less than 0.05 indicates statistical significance using the Mann-Whitney U-test. ^*^P < 0.05, ^**^P < 0.01, ^***^P < 0.001.

We further performed ELISA to clarify the changes in the ALB, ARHGDIB, C3, FCN2, and LPA levels during anti-TB treatment in 57 untreated patients (0 month), 53 2-month treated patients (2 months), and 59 cured patients (>6 months). Significant differences were observed at the three time points for the five proteins (*P* < 0.001, *P* < 0.001, *P* = 0.011, *P* = 0.030, and *P* = 0.014, respectively). Additionally, linear trends were observed in the five proteins at the three time points (*P* = 0.008, *P* < 0.001, *P* = 0.008, *P* = 0.004, and *P* = 0.005, respectively). Serum levels of ALB and FCN2 were altered after 2 months of treatment (*P* < 0.001 and *P* = 0.030, respectively) (Fig. 3). All untreated TB patients were separated by age, gender, sputum smear results, lung lesions, and chest X-ray results. Significantly increased ARHGDIB levels and reduced FCN2 levels were observed in untreated smear-negative TB patients compared with smear-positive TB patients (*P* < 0.001 and *P* = 0.016) (Table 3). However, no such differences were observed in cured TB patients.

### Clinical data analysis

The clinical data analysis revealed significant differences between untreated TB patients and controls in levels of the following (*P* < 0.05): total protein, albumin, globulin, albumin/globulin, total cholesterol, triglyceride, high-density lipoprotein cholesterol (HDL-C), low-density lipoprotein cholesterol (LDL-C), lipoprotein (a), apolipoprotein A1 (APOA1), apolipoprotein B (APOB), C-reactive protein, prealbumin, IgG, IgA, IgM, and complement 4. Similar to the ELISA results, total protein, albumin, globulin, albumin/globulin, lipoprotein (a), and glucose levels significantly differed between the cured and untreated TB patients (*P* < 0.05) (Table 4). We further observed significant negative correlations between ALB and C-reactive protein, and ALB and IgA levels (r = −0.300, *P* = 0.048; and r = −0.485, *P* = 0.042, respectively); a significant positive correlation between LPA and total cholesterol levels (r = 0.345, *P* = 0.025); and significantly negative correlations between C3 and total protein, albumin, total cholesterol, and APOA1 levels (r = −0.473, *P* = 0.002; r = −0.471, *P* = 0.002; r = −0.331, *P* = 0.035; and r = −0.388, *P* = 0.012, respectively) by the Spearman correlation analysis.

### ROC analysis

**Figure 4:**
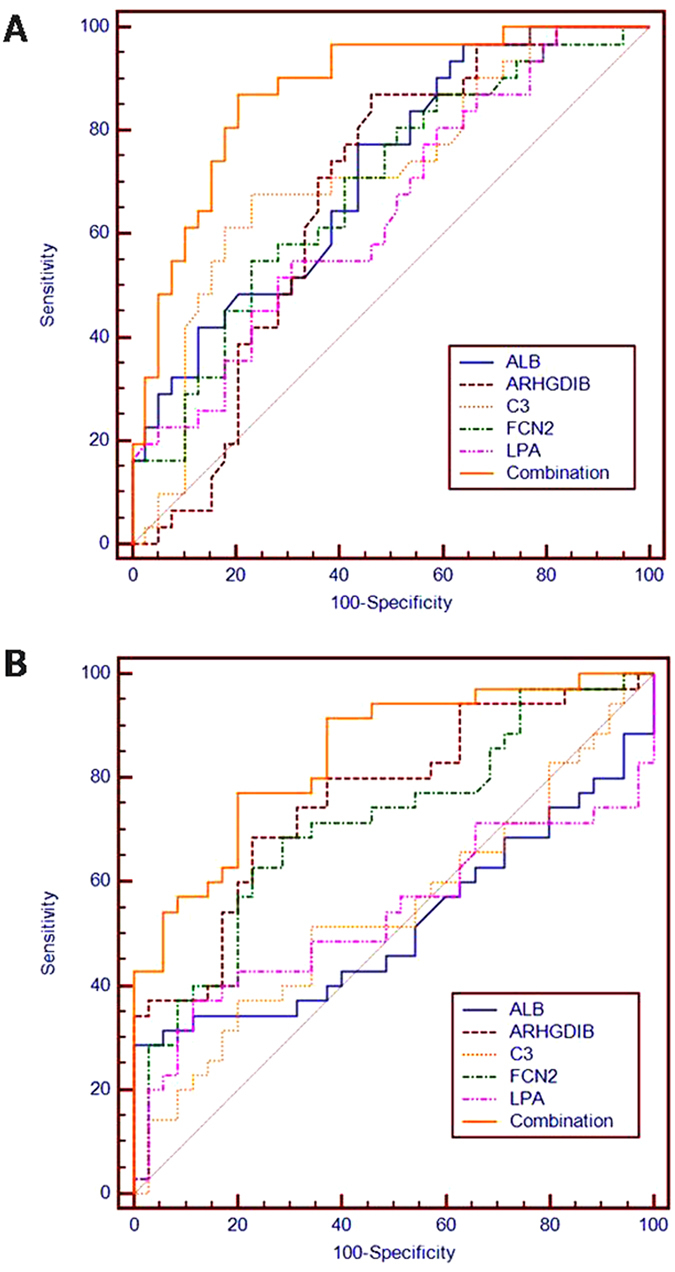
The receiver operation characteristics (ROC) curve analyses. ROC curve analyses of the ALB, ARHGDIB, C3, FCN2, and LPA levels as well as the combination. (**A**) A model to discriminate cured tuberculosis patients from untreated tuberculosis patients. (**B**) A model to discriminate sputum-positive patients from sputum-negative patients.

We performed receiver operating characteristic (ROC) analysis to evaluate the sensitivity and specificity of the five proteins, and the areas under the curve (AUC) were 0.716 for ALB, 0.674 for ARHGDIB, 0.714 for C3, 0.689 for FCN2, and 0.646 for LPA. Next, we developed a model using forward stepwise multivariate regression: Logit (p) = −0.1617+0.04934*(ALB) −0.0189*(ARHGDIB)-0.001431*(C3) −0.06697*(FCN2) +0.003786*(LPA). When the 5 proteins served as a panel, the sensitivity, specificity, and AUC increased to 87.10%, 79.49%, and 0.876, respectively (Fig. 4A). Because we found a significant difference between sputum-positive and sputum-negative patients, we established another model to discriminate between these patients: Logit (p′) = 0.7113–0.03258*(ARHGDIB)+0.00165*(C3) +0.05926*(FCN2). The sensitivity, specificity, and AUC for this model were 77.14%, 65.71%, and 0.845, respectively (Fig. 4B).

## Discussion

China has the world’s second largest TB epidemic^1^ with 4.99 million active TB cases, including 720,000 smear-positive cases and 1.29 million culture-positive cases^12^. However, due to the lack of rapid and efficient methods for determining cured TB, 14% of the patients are discharged without being fully cured and are vulnerable to relapse. The relapse rate for TB is 5.3% globally^1^, whereas the rate is 11.8% in China^12^. In addition, 20.5% of previously treated TB cases are estimated to develop multi-drug resistant TB, which is greater than newly treated cases (3.5%)^3^, and may increase transmission potential.

Previously reported 2-month TB treatment biomarkers, such as coagulation factor V, thrombospondin-4, and alpha-1-antitrypsin^10^,^13^, and previously reported diagnostic biomarkers, such as apolipoprotein A-II^14^ and fibrinogen beta chain^15^, were also identified by iTRAQ-2DLC-MS/MS analysis in cured and untreated TB patients in our study. Although coronin-1A (fold change = 0.60, *P* < 0.05), proteasome subunit alpha type-5 (fold change = 0.32, *P* < 0.05), and MAP/microtubule affinity-regulating kinase 4 (fold change = 4.00, *P* < 0.05) function in T-cell activation^16^, apoptosis^17^, and the positive regulation of programmed cell death^18^, commercial ELISA kits for these proteins are not available. Further studies on these unselected proteins may help us identify more effective diagnostic strategies for the determination of cured TB. ELISA revealed significantly increased serum ALB and LPA levels and significantly reduced ARHGDIB, C3, and FCN2 levels in cured TB patients compared with untreated ones, including both smear-positive (Fig. 2) and smear-negative patients.

ALB is reduced during inflammatory states. In this study, ALB levels were reduced in untreated TB patients compared with controls (*P* < 0.001). An epidemiological follow-up study found that people with low serum ALB levels were susceptible to TB (*P* = 0.006)^19^, whereas TB patients with low serum ALB levels were vulnerable to death (*P* < 0.001)^20^, indicating that ALB levels of patients could influence the pathogenesis and prognosis of TB. LPA is a hydrophilic glycoprotein that can be assembled with LDL^21^ and potentially exhibits coagulated function^22^. Apolipoproteins serve as biomarkers for the diagnosis and treatment of TB^14^,^23^,^24^,^25^. We also found that the total cholesterol, triglyceride, HDL-C, LDL-C, lipoprotein (a), APOA1, and APOB levels were significantly altered in TB patients (Table 4), indicating that lipid metabolism biomarkers might be used as indicators for anti-TB treatment.

Rho GDP dissociation inhibitor 2 (ARHGDIB, LyGDI) not only plays a role in inflammation and immunity^26^,^27^,^28^, but also affects cell invasion by regulating the extracellular matrix^29^,^30^,^31^. In the present study, ARHGDIB increased significantly in untreated TB patients compared with controls (*P* = 0.003) and decreased in cured TB patients (*P* < 0.001). No significant difference was noted between cured TB patients and controls. Therefore, we suspect that ARHGDIB might affect the formation of TB granulomas through the regulation of extracellular matrix proteins, resulting in its increase in untreated TB patients followed by a reduction in cured TB patients. Moreover, ARHGDIB levels were significantly reduced in smear-positive patients compared with smear-negative patients (*P* < 0.001) (Table 3). We suggest that smear-negative patients infected with lower amounts of bacteria^32^, which might exhibit more proliferative pathological changes, and lead to a higher level of ARHGDIB.

The complement system has been seriously studied in TB studies^23^,^33^,^34^,^35^. As a part of the complement system, C3 is potentially related to TB pathogenesis^34^ and anti-TB treatment^35^. Our study revealed a C3 level reduction in cured TB patients (*P* = 0.035). FCN2 is involved in the immune defense. This protein binds to pathogen-associated molecular patterns (PAMPs) on the pathogen surface and initiates the complement lectin cascade, thereby clearing the pathogens^36^,^37^. Eisen *et al.*^38^ proposed that FCN2 is associated with inflammatory changes in the respiratory system, which is supported by our findings. Faik *et al.*^39^ found that FCN2 levels decreased significantly after malaria treatment (*P* < 0.0001). Our study also revealed decreased FCN2 levels after anti-TB treatment. Therefore, we suspect that the lower levels of C3 and FCN2 in cured TB patients (*P* = 0.035, *P* = 0.018, Fig. 3) might be due to the alleviation of complement system activation. In addition, FCN2 levels were significantly increased in smear-positive patients compared with smear-negative patients (*P* = 0.016, Table 3). We assumed that smear-negative patients had a higher proportion of latent infections^40^, resulting in limited complement system activation and reduced FCN2 levels.

In summary, ALB and LPA levels increased significantly in cured TB patients, whereas ARHGDIB, C3, and FCN2 levels decreased significantly. This finding might be due to the improved inflammation and lipid metabolism status as well as eased immune system and complement system activation status. Therefore, ALB, LPA, ARHGDIB, C3, and FCN2 levels might serve as potential biomarkers for cured TB. In addition, significant linear trends and increased levels of ALB and FCN2 were observed in 2-month treated TB patients (*P* < 0.001 and *P* = 0.030, Fig. 3), indicating the predictive value of ALB and FCN2 levels for treatment outcomes.

Studies have demonstrated that a diagnostic model established by a combination of markers exhibits increased sensitivity and specificity compared with a single marker model^14^,^41^. Our study established a model with a sensitivity of 87.10%, a specificity of 79.49%, and an AUC of 0.876. The sensitivity and specificity were not only higher than those for single proteins but were also higher than those of the sputum smear and sputum culture; thus, the model was more accurate for cured TB determination. The lack of efficacy evaluation markers largely hinders the development of new anti-TB drugs and therapies^8^,^42^. Our study established a combination model to provide the experimental basis for evaluating the efficacy of anti-TB drugs.

## Methods

### Patients and Control Subjects

The complete details of the entire study design and procedures involved were in accordance with the Declaration of Helsinki. This study was approved by the Ethics Committee of the Faculty of Medicine Zhejiang University, China. Written informed consent was obtained from all subjects before blood sampling. The methods used were carried out in accordance with approved guidelines and regulations.

Blood was drawn into regular bottles in the morning from newly diagnosed TB patient at three time points (before anti-TB therapy, after the intensive phase, and upon cure) between November 2013 and November 2014 at the Sixth Hospital of Shaoxing and the First Hospital of Jiaxing. Pulmonary TB patients were diagnosed according to the diagnostic criteria of the Ministry of Health, China^43^. All patients met one of the following pulmonary TB diagnostic criteria: (1) a positive sputum examination (smear or culture); (2) a negative sputum examination and a chest X-ray and CT revealing evidence typical of active TB; (3) a pathological diagnosis of TB in lung specimens; (4) suspected pulmonary TB after clinical follow-up and X-ray observations after excluding other lung diseases; and (5) clinical elimination of other causes of pleural effusion and a diagnosis of tuberculosis pleurisy. We recruited both smear positive and negative patients. Standard TB therapy consists of rifampin, isoniazid, pyrazinamide and ethambutol for the first 2 months followed by rifampin and isoniazid for an additional 4 to 6 months^2^. Patients with extra-pulmonary TB, malignancies, chronic disease, autoimmune diseases, or HIV infection were not included in the study. Fasting blood samples were drawn from healthy controls at the Zhejiang Hospital. The samples were stored at −80 °C for further analysis. Data including age, gender and clinical examination findings of TB patients and controls were collated into databases separately by different time points. In total, 122 untreated TB patients, 91 2-month treated TB patients, 59 cured TB patients, and 122 healthy controls were enrolled in the study.

A total of 57 untreated TB subjects (33 males, 24 females; aged 18–64 years; mean age 38.03 ± 14.94 years), 53 2-month treated TB subjects (27 males, 26 females; aged 18–65 years; mean age 35.55 ± 14.66 years), 59 cured TB subjects (37 males, 22 females; aged 18–75 years; mean age 40.97 ± 15.55 years), and 60 healthy subjects (35 males, 25 females; aged 24–73 years; mean age 41.55 ± 13.16 years) underwent ELISA testing (Supplementary Table S2).

### iTRAQ-2DLC-MS/MS

To increase the precision and accuracy of the data in the proteomics study^44^, equal amounts of 10 different samples were mixed to produce a sample group. In addition, 10 samples were randomly divided into two pools as biological replicates. Then, iTRAQ-labeled sample pools were generated.

High-abundance serum proteins, such as albumin, IgG, and haptoglobin, were removed using the Human 14 Multiple Affinity Removal System (Agilent Technologies, Santa Clara, CA, USA). Next, the proteins were concentrated and desalted^14^. A total of 100 μg of protein from each group were soaked in ice-cold acetone and then centrifuged. The subsidence samples were reduced and blocked. The samples were then digested with trypsin at 37°C overnight. Finally, iTRAQ reagents (Applied Biosystems, Foster city, CA, USA) were labeled as follows: the control group, iTRAQ reagent 113, 117; the untreated smear-positive pulmonary TB group, iTRAQ reagent 114, 118; and the cured pulmonary TB group, iTRAQ reagent 115, 119. The six sample groups were mixed, desalted, and dried. The iTRAQ-labeled peptides were separated using a polysulfoethyl column (2.1 × 100 mm, 5 μm, 200 Ǻ; Nest Group, Inc., Southborough, MA, USA) with strong cation exchange (SCX) chromatography^45^. A total of ten SCX components were collected, concentrated, and dissolved. Samples were subsequently loaded onto the ZORBAX 300SB-C18 column (5 μm, 200 Ǻ, 0.1 × 150 mm; Microm, Auburn, CA, USA). The components produced by SCX chromatography were subjected to MS analysis twice. The ratio of the peak area of the iTRAQ reporter ion reflected the relative abundance of the peptide and protein^46^,^47^. Protein identification and quantification were performed using the ProteinPilotTM version 4.2 software (Applied Biosystems). The ProGroup algorithm was used to identify peptides. MS/MS data were searched against the Human International Protein Index database (version 3.87) with parameter settings as described previously^14^,^46^,^47^. To reduce false positive results, a strict cutoff for protein identification was applied with the unused ProtScore >1.3 and at least one peptide with a 95% confidence limit^48^,^49^. The protein expression ratio between the two groups (<0.60 or >1.50) was considered significant. The cellular component, molecular function, and biological process were analyzed by the Gene Ontology database, whereas KEGG pathway analysis was performed using the KEGG database (false discovery rate <1.00%). The protein–protein network was analyzed by STRING software (http://string-db.org/).

### ELISA methods

The albumin human ELISA kit (Abcam, Cambridge, MA, USA; SwissProt: P02768), the lipoprotein A (APOA, LPA) human ELISA kit (Abcam, Cambridge, MA, USA; SwissProt: P08519), the human rho GDP-dissociation inhibitor 2 (ARHGDIB) ELISA kit (Cusabio Biotech. Co., LTD, China; SwissProt: P52566), the complement C3 human ELISA kit (Abcam, Cambridge, MA, USA; SwissProt: P01024), and the human Ficolin-2 (FCN2) ELISA kit (Cusabio Biotech. Co., LTD, China; SwissProt: Q15485) were used to detect protein levels in the serum. The protein concentration of 57 untreated TB patients, 53 2-month treated TB patients, 59 cured TB patients and 60 healthy controls were measured according to the manufacturer’s instructions. Serum samples were diluted with dilution factors of 1:10,000, 1:800, 1:4,000, and 1:20,000 for ALB, C3, FCN2, and LPA, respectively. ELISAs were performed according to the instructions of each kit.

### Statistical Analysis

Parametric data are presented as the mean ± standard deviation (SD), whereas nonparametric data are presented as the median ± interquartile range (IQR). *P-*values < 0.05 are considered statistically significant by the SPSS software (version 16.0, Chicago, IL). The parametric data were tested using the chi-square test for the composition ratios and *t*-tests and one-way analysis of variance (ANOVA) for means. Nonparametric analysis was performed using the Mann–Whitney U-test for two groups and the Kruskal-Wallis H-test for three or more groups. The test for linear trends was performed to examine the trend of protein expression during treatment, whereas Spearman’s correlation method was performed to determine the association between two different parameters. For each protein, a ROC curve was generated. During model construction, the diagnostic score of untreated TB patients was set as 0, whereas that of cured TB patients was set as 1. In the other model, the score of sputum-positive patients were 1, and the score of sputum-negative patients were 0. To increase the diagnostic accuracy of the combined serum proteins, multiple logistic regression analysis was performed. ROC curves and logistic regression models were calculated using MedCalc Software (Version 12.4.2.0, Belgium).

## Additional Information

**How to cite this article**: Wang, C. *et al.* Screening and identification of five serum proteins as novel potential biomarkers for cured pulmonary tuberculosis. *Sci. Rep.* **5**, 15615; doi: 10.1038/srep15615 (2015).

## Supplementary Material

Supplementary Information

## Figures and Tables

**Table 1:** Up-regulated proteins and their expression levels among cured and untreated tuberculosis patients and controls quantified by iTRAQ-2DLC-MS/MS.

Protein ID	Protein name	Ratios		
		Cured TB/Untreated TB	Untreated TB/Controls	Cured TB/Controls
P01599	Ig kappa chain V-I region Gal	12.39	0.52	6.44
P02768	Serum albumin	8.11	0.02	0.20
P62805	Histone H4	7.79	0.09	–
P08519	Apolipoprotein(a)	3.98	0.38	–
P02647	Apolipoprotein A-I	3.80	0.44	1.67
P01834	Ig kappa chain C region	2.68	0.46	–
O60462	Neuropilin-2	2.55	0.52	–
P02652	Apolipoprotein A-II	2.38	0.43	–
P01876	Ig alpha-1 chain C region	2.34	0.31	–
P04432	Ig kappa chain V-I region Daudi	2.13	0.48	–
Q13449	Limbic system-associated membrane protein	1.71	0.54	–
P12259	Coagulation factor V	1.67	0.52	–
P22105	Tenascin-X	1.54	0.58	–
P30101	Protein disulfide-isomerase A3	1.53	0.51	–
P10909	Clusterin	1.53	0.52	–

TB: tuberculosis; −: ratio between 0.60 and 1.50.

**Table 2:** Down-regulated proteins and their expression levels among cured and untreated tuberculosis patients and controls quantified by iTRAQ-2DLC-MS/MS.

Protein ID	Protein name	Ratios		
		Cured TB/Untreated TB	Untreated TB/Controls	Cured TB/Controls
P05109	Protein S100-A8	0.27	4.20	–
P02741	C-reactive protein	0.28	20.71	5.79
P52566	Rho GDP-dissociation inhibitor 2	0.28	1.70	0.48
P02763	Alpha-1-acid glycoprotein 1	0.31	15.32	4.82
P0DJI8	Serum amyloid A-1 protein	0.32	6.84	2.19
Q05682	Caldesmon	0.32	1.89	–
P28066	Proteasome subunit alpha type-5	0.32	1.93	–
P01024	Complement C3	0.40	1.83	–
P01009	Alpha-1-antitrypsin	0.41	2.85	–
P01011	Alpha-1-antichymotrypsin	0.41	2.51	–
P09960	Leukotriene A-4 hydrolase	0.42	2.28	–
P06702	Protein S100-A9	0.43	1.74	–
P19652	Alpha-1-acid glycoprotein 2	0.47	7.59	3.54
P07477	Trypsin-1	0.50	1.86	–
P08637	Low affinity immunoglobulin gamma Fc region receptor III-A	0.51	1.77	–
P35527	Keratin, type I cytoskeletal 9	0.51	4.50	2.29
P01880	Ig delta chain C region	0.55	6.43	3.54
Q15485	Ficolin-2	0.56	2.74	1.53
P13796	Plastin-2	0.59	1.99	–
P31146	Coronin-1A	0.60	2.93	1.76

TB: tuberculosis; −: ratio between 0.60 and 1.50.

**Table 3:** Average serum levels of proteins according to the clinical characteristics of the untreated tuberculosis patients.

Clinical characteristics (Cases)	ALB (mg/mL)	*P*^a^	ARHGDIB (pg/mL)	*P*^a^	C3 (μg/mL)	*P*^a^	FCN2 (μg/mL)	*P*^a^	LPA (μg/mL)	*P*^a^
**Age**		0.772		0.055		0.227		0.684		0.418
18–29 (21)	48.17 ± 32.71		80.11 ± 44.69		576.00 ± 781.00		18.78 ± 18.66		131.08 ± 118.21	
30–49 (21)	49.57 ± 27.74		114.25 ± 93.23		558.80 ± 330.60		13.77 ± 20.77		126.48 ± 50.72	
≥50 (15)	42.20 ± 28.97		126.82 ± 79.34		747.20 ± 333.60		14.30 ± 12.64		153.39 ± 46.36	
**Gender**		0.948		0.164		0.755		0.579		0.389
Male (33)	47.35 ± 31.20		87.88 ± 92.27		614.40 ± 497.20		15.28 ± 14.69		135.15 ± 92.65	
Female (24)	47.82 ± 30.96		110.33 ± 82.08		607.20 ± 582.40		13.85 ± 19.24		146.67 ± 89.79	
**Sputum smear**		0.263		<0.001^***^		0.894		0.016^*^		0.495
Negative (21)	48.49 ± 18.76		147.87 ± 52.89		608.80 ± 325.20		10.32 ± 8.54		148.27 ± 38.30	
Positive (36)	39.55 ± 31.14		71.16 ± 57.24		622.40 ± 860.00		19.65 ± 22.03		128.52 ± 159.35	
**Lung lesion**		0.218		0.720		0.107		0.800		0.059
Single (21)	52.59 ± 28.29		89.22 ± 44.35		512.80 ± 483.20		16.18 ± 15.14		156.72 ± 119.18	
Double (36)	44.19 ± 28.82		112.51 ± 97.18		622.40 ± 921.20		14.42 ± 19.91		126.48 ± 51.67	
**Chest X-ray**		0.078		0.379		0.229		0.196		0.235
Non-cavity (47)	48.74 ± 26.96		88.27 ± 91.89		584.00 ± 460.00		14.18 ± 18.33		144.26 ± 96.74	
Cavity (10)	28.67 ± 22.72		118.76 ± 48.04		931.60 ± 1116.20		20.76 ± 20.73		113.04 ± 54.11	

All data are presented as the median ± IQR. ^a^*P*-value among groups, the Mann–Whitney U-test is used for two groups and the Kruskal-Wallis H-test is used for three groups. ^*^P < 0.05, ^***^P < 0.001.

**Table 4:** Clinical data of the pulmonary tuberculosis patients and controls.

	Controls (N = 122)	Untreated TB (N = 122)	2-month treated TB (N = 91)	Cured TB (N = 59)	*P*^a^		
					Untreated TB *vs.*Controls	Untreated TB *vs.* 2-month treated TB	Untreated TB *vs.* Cured TB
Total protein (g/L)	72.02 ± 3.52	70.68 ± 6.11	73.60 ± 6.19	72.76 ± 6.01	0.038^*^	0.001^**^	0.032^*^
Albumin (g/L)	46.16 ± 2.08	41.06 ± 4.79	43.84 ± 4.13	45.17 ± 4.74	<0.001^***^	<0.001^***^	<0.001^***^
Globulin (g/L)	25.88 ± 3.00	29.63 ± 4.99	29.77 ± 5.38	27.59 ± 4.51	<0.001^***^	0.848	0.009^**^
A/G	1.81 ± 0.23	1.43 ± 0.30	1.51 ± 0.34	1.68 ± 0.30	<0.001^***^	0.066	<0.001^***^
Total cholesterol (mmol/L)	4.95 ± 0.99	3.73 ± 0.82	4.01 ± 1.04	3.76 ± 1.08	<0.001^***^	0.193	0.904
Triglyceride (mmol/L)	1.52 ± 1.04	1.03 ± 0.54	1.07 ± 0.48	0.89 ± 0.33	<0.001^***^	0.644	0.291
HDL-C (mmol/L)	1.31 ± 0.33	1.22 ± 0.39	1.32 ± 0.41	1.22 ± 0.49	0.037^*^	0.095	0.997
LDL-C (mmol/L)	2.95 ± 0.79	2.28 ± 0.60	2.38 ± 0.85	2.26 ± 0.78	<0.001^***^	0.329	0.919
Lipoprotein (a) (mg/L)	210.11 ± 161.70	151.55 ± 101.92	187.54 ± 152.94	215.23 ± 88.96	0.001^**^	0.068	0.040^*^
APOA1 (g/L)	1.24 ± 0.27	1.13 ± 0.26	1.26 ± 0.27	1.20 ± 0.30	0.001^**^	0.001^**^	0.293
APOB (g/L)	0.93 ± 0.47	0.83 ± 0.18	0.84 ± 0.27	0.83 ± 0.25	0.044^*^	0.904	0.889
Glucose (mmol/L)	5.16 ± 1.05	5.02 ± 1.14	5.16 ± 1.36	6.05 ± 2.30	0.329	0.488	0.005^**^
CRP (mg/L)	0.91 ± 1.34	21.21 ± 31.08	10.53 ± 18.59	22.30 ± 25.56	0.001^**^	0.025^*^	0.910
Prealbumin (g/L)	0.22 ± 0.06	0.17 ± 0.07	0.18 ± 0.08	0.19 ± 0.06	<0.001^***^	0.665	0.532
IgG (g/L)	12.74 ± 2.21	14.31 ± 3.78	15.61 ± 3.44	14.04 ± 5.96	<0.001^***^	0.058	0.878
IgA (g/L)	2.12 ± 0.90	3.02 ± 1.43	3.08 ± 1.45	2.05 ± 1.87	<0.001^***^	0.842	0.147
IgM (g/L)	0.99 ± 0.48	1.41 ± 0.67	1.34 ± 0.87	1.04 ± 1.58	<0.001^***^	0.599	0.279
Complement 4 (mg/L)	219.51 ± 61.06	335.08 ± 99.88	298.38 ± 87.30	296.25 ± 116.12	<0.001^***^	0.041^*^	0.450

All data are presented as the mean ± SD. A/G: albumin/globulin ratio; HDL-C: high-density lipoprotein cholesterol; LDL-C: low-density lipoprotein cholesterol; APOA1: apolipoprotein A1; APOB: apolipoprotein B; CRP: C-reactive protein. ^a^*P*-value between two groups using the *t*-test. ^*^P < 0.05, ^**^P < 0.01, ^***^P < 0.001.
