# Supplementary material for: Screening and identification of five serum proteins as novel potential biomarkers for cured pulmonary tuberculosis
Source: Sci Rep. 2015 Oct 26;5:15615. doi: 10.1038/srep15615 (PMC4620482; doi:10.1038/srep15615)
Supplement: Supplementary Information [file srep15615-s1.doc]

**Supplementary Information**

Screening and identification of five serum proteins as novel potential biomarkers for cured pulmonary tuberculosis

Chong Wang^1^, Li-Liang Wei^2^, Li-Ying Shi^3^, Zhi-Fen Pan^4^, Xiao-Mei Yu^3^, Tian-Yu Li^1^, Chang-Ming Liu^1^, Ze-Peng Ping^1^, Ting-Ting Jiang^1^, Zhong-Liang Chen^1^, Lian-Gen Mao^1^, Zhong-Jie Li^1^, Ji-Cheng Li^1*^

**Supplementary Table S1.** Differentially expressed proteins and their expression levels between cured and untreated tuberculosis patients quantified by iTRAQ-2DLC-MS/MS

| Protein ID | Protein name | | | | | Ratio |
| --- | --- | --- | --- | --- | --- | --- |
| **Increased proteins** | |  | |  | | |
| P01599 | Ig kappa chain V-I region Gal | | | | | 12.39 |
| P01613 | Ig kappa chain V-I region Ni | | | | | 8.23 |
| P02768 | Serum albumin | | | | | 8.11 |
| P62805 | Histone H4 | | | | | 7.79 |
| P10124 | Serglycin | | | | | 7.20 |
| P24821 | Tenascin | | | | | 6.27 |
| Q96L34 | MAP/microtubule affinity-regulating kinase 4 | | | | | 4.00 |
| P08519 | Apolipoprotein(a) | | | | | 3.98 |
| P02647 | Apolipoprotein A-I | | | | | 3.80 |
| Q86VB7 | Scavenger receptor cysteine-rich type 1 protein M130 | | | | | 3.13 |
| Q92614 | Unconventional myosin-XVIIIa | | | | | 3.04 |
| P01344 | Insulin-like growth factor II | | | | | 2.82 |
| P01834 | Ig kappa chain C region | | | | | 2.68 |
| O60462 | Neuropilin-2 | | | | | 2.55 |
| P49747 | Cartilage oligomeric matrix protein | | | | | 2.49 |
| P02652 | Apolipoprotein A-II | | | | | 2.38 |
| P14543 | Nidogen-1 | | | | | 2.37 |
| P01876 | Ig alpha-1 chain C region | | | | | 2.34 |
| P06314 | Ig kappa chain V-IV region B17 | | | | | 2.27 |
| P04432 | Ig kappa chain V-I region Daudi | | | | | 2.13 |
| P02042 | Hemoglobin subunit delta | | | | | 2.11 |
| P35443 | Thrombospondin-4 | | | | | 2.11 |
| P01860 | Ig gamma-3 chain C region | | | | | 2.08 |
| P04208 | Ig lambda chain V-I region WAH | | | | | 2.05 |
| P0CG05 | Ig lambda-2 chain C regions | | | | | 2.01 |
| P43121 | Cell surface glycoprotein MUC18 | | | | | 1.98 |
| P04433 | Ig kappa chain V-III region VG (Fragment) | | | | | 1.92 |
| P20742 | Pregnancy zone protein | | | | | 1.87 |
| P01833 | Polymeric immunoglobulin receptor | | | | | 1.85 |
| P81605 | Dermcidin | | | | | 1.76 |
| P01743 | Ig heavy chain V-I region HG3 | | | | | 1.75 |
| P35542 | Serum amyloid A-4 protein | | | | | 1.74 |
| P02656 | Apolipoprotein C-III | | | | | 1.72 |
| Q13449 | Limbic system-associated membrane protein | | | | | 1.71 |
| Q15063 | Periostin | | | | | 1.68 |
| Q9Y646 | Carboxypeptidase Q | | | | | 1.67 |
| P12259 | Coagulation factor V | | | | | 1.67 |
| P04070 | Vitamin K-dependent protein C | | | | | 1.63 |
| P01701 | Ig lambda chain V-I region NEW | | | | | 1.60 |
| O00187 | Mannan-binding lectin serine protease 2 | | | | | 1.58 |
| P01033 | Metalloproteinase inhibitor 1 | | | | | 1.56 |
| P22105 | Tenascin-X | | | | | 1.54 |
| P30101 | Protein disulfide-isomerase A3 | | | | | 1.53 |
| P02654 | Apolipoprotein C-I | | | | | 1.53 |
| P10909 | Clusterin | | | | | 1.53 |
| P01766 | Ig heavy chain V-III region BRO | | | | | 1.52 |
| P01598 | Ig kappa chain V-I region EU | | | | | 1.52 |
| Q12907 | Vesicular integral-membrane protein VIP36 | | | | | 1.51 |
| Q9P232 | Contactin-3 | | | | | 1.51 |
| P12111 | Collagen alpha-3(VI) chain | | | | | 1.51 |
| P10586 | Receptor-type tyrosine-protein phosphatase F | | | | | 1.50 |
| **Decreased proteins** | | |  | |  | |
| O95810 | Serum deprivation-response protein | | | | | 0.17 |
| P62328 | Thymosin beta-4 | | | | | 0.21 |
| P67936 | Tropomyosin alpha-4 chain | | | | | 0.23 |
| P05109 | Protein S100-A8 | | | | | 0.27 |
| P02775 | Platelet basic protein | | | | | 0.28 |
| P02741 | C-reactive protein | | | | | 0.28 |
| P52566 | Rho GDP-dissociation inhibitor 2 | | | | | 0.28 |
| P02763 | Alpha-1-acid glycoprotein 1 | | | | | 0.31 |
| P0DJI8 | Serum amyloid A-1 protein | | | | | 0.32 |
| Q05682 | Caldesmon | | | | | 0.32 |
| P28066 | Proteasome subunit alpha type-5 | | | | | 0.32 |
| P37802 | Transgelin-2 | | | | | 0.36 |
| P10645 | Chromogranin-A | | | | | 0.39 |
| P01024 | Complement C3 | | | | | 0.40 |
| P01009 | Alpha-1-antitrypsin | | | | | 0.41 |
| P01011 | Alpha-1-antichymotrypsin | | | | | 0.41 |
| P09960 | Leukotriene A-4 hydrolase | | | | | 0.42 |
| P06702 | Protein S100-A9 | | | | | 0.43 |
| P07996 | Thrombospondin-1 | | | | | 0.44 |
| P02776 | Platelet factor 4 | | | | | 0.45 |
| P19652 | Alpha-1-acid glycoprotein 2 | | | | | 0.47 |
| P31431 | Syndecan-4 | | | | | 0.48 |
| P07477 | Trypsin-1 | | | | | 0.50 |
| P08637 | Low affinity immunoglobulin gamma Fc region receptor III-A | | | | | 0.51 |
| P35527 | Keratin, type I cytoskeletal 9 | | | | | 0.51 |
| P20774 | Mimecan | | | | | 0.54 |
| P02675 | Fibrinogen beta chain | | | | | 0.55 |
| P01880 | Ig delta chain C region | | | | | 0.55 |
| Q15485 | Ficolin-2 | | | | | 0.56 |
| P63104 | 14-3-3 protein zeta/delta | | | | | 0.58 |
| P13796 | Plastin-2 | | | | | 0.59 |
| P21333 | Filamin-A | | | | | 0.59 |
| Q9UJJ9 | N-acetylglucosamine-1-phosphotransferase subunit gamma | | | | | 0.59 |
| P31146 | Coronin-1A | | | | | 0.60 |

**Supplementary Table S2.** Characteristics of the tuberculosis patients and controls

|  | Untreated TB (N=57) | 2-month treated TB (N=53) | Cured TB (N=59) | Controls (N=60) | *P* |
| --- | --- | --- | --- | --- | --- |
| Age, years range (mean ± SD) | 18-64 (38.03±14.94) | 18-65 (35.55±14.66) | 18-75 (40.97±15.55) | 24-73 (41.55±13.16) | 0.110^a^ |
| Gender: female, no. (%) | 24 (42.11) | 26 (49.06) | 22 (37.29) | 25 (41.67) | 0.658^b^ |
| Body mass index (mean ± SD) | 20.03±2.38 | 20.82±2.29 | 21.41±2.33 | 22.21±3.75 | 0.211^a^ |
| Presence of TB history of relatives, no. (%) | 10 (17.54) | 9 (16.98) | 10 (16.95) | 6 (10.00) | 0.623^b^ |
| BCG vaccination, no. (%) | 20 (35.09) | 19 (35.85) | 19 (32.20) | 30 (50.00) | 0.189^b^ |

N: number of subjects; TB: tuberculosis. ^a^*P*-value among four groups from one-way ANOVA. ^b^*P*-value among four groups from the chi-square test.
